# Differential Roles for STIM1 and STIM2 in Store-Operated Calcium Entry in Rat Neurons

**DOI:** 10.1371/journal.pone.0019285

**Published:** 2011-04-26

**Authors:** Joanna Gruszczynska-Biegala, Pawel Pomorski, Marta B. Wisniewska, Jacek Kuznicki

**Affiliations:** 1 Laboratory of Neurodegeneration, International Institute of Molecular and Cell Biology, Warsaw, Poland; 2 Department of Biochemistry, Nencki Institute of Experimental Biology, Warsaw, Poland; Cornell University, United States of America

## Abstract

The interaction between Ca^2+^ sensors STIM1 and STIM2 and Ca^2+^ channel-forming protein ORAI1 is a crucial element of store-operated calcium entry (SOCE) in non-excitable cells. However, the molecular mechanism of SOCE in neurons remains unclear. We addressed this issue by establishing the presence and function of STIM proteins. Real-time polymerase chain reaction from cortical neurons showed that these cells contain significant amounts of *Stim1* and *Stim2* mRNA. Thapsigargin (TG) treatment increased the amount of both endogenous STIM proteins in neuronal membrane fractions. The number of YFP-STIM1/ORAI1 and YFP-STIM2/ORAI1 complexes was also enhanced by such treatment. The differences observed in the number of STIM1 and STIM2 complexes under SOCE conditions and the differential sensitivity to SOCE inhibitors suggest their distinct roles. Endoplasmic reticulum (ER) store depletion by TG enhanced intracellular Ca^2+^ levels in loaded with Fura-2 neurons transfected with YFP-STIM1 and ORAI1, but not with YFP-STIM2 and ORAI1, which correlated well with the number of complexes formed. Moreover, the SOCE inhibitors ML-9 and 2-APB reduced Ca^2+^ influx in neurons expressing YFP-STIM1/ORAI1 but produced no effect in cells transfected with YFP-STIM2/ORAI1. Moreover, in neurons transfected with YFP-STIM2/ORAI1, the increase in constitutive calcium entry was greater than with YFP-STIM1/ORAI1. Our data indicate that both STIM proteins are involved in calcium homeostasis in neurons. STIM1 mainly activates SOCE, whereas STIM2 regulates resting Ca^2+^ levels in the ER and Ca^2+^ leakage with the additional involvement of STIM1.

## Introduction

Store-operated calcium entry (SOCE), also referred to as capacitative calcium entry (CCE), is a phenomenon that has been well characterized in non-excitable cells. In these cells, the Ca^2+^ signal usually originates from the induction of metabotropic receptors, leading to the production of IP_3_ by plasma membrane-located phospholipase and release of Ca^2+^ from intracellular stores by activity of IP_3_ receptors. This early stage is followed by SOCE, which relies on extracellular Ca^2+^ influx through the SOC channels present in the plasma membrane (PM) and is tightly regulated by Ca^2+^ concentration in the endoplasmic reticulum (ER) [1], [2]. This influx allows refilling of the ER with Ca^2+^ ions after their IP_3_-dependent release to the cytoplasm [3], [4]. The known proteins involved in this process are sensors of Ca^2+^ levels in the ER, including STIM1 and STIM2 [5], [6], and the Ca^2+^ channel-forming protein ORAI1 in the plasma membrane [7], [8], [9]. The interaction between STIMs and ORAI is a crucial element of calcium homeostasis in non-excitable cells and leads to the formation of complexes visible in fluorescent microscopy as so-called “puncta” (reviewed by [10]). Calcium entry into the cytoplasm is replenished in the ER by the activity of the Ca^2+^ adenosine triphosphatase (ATPase) of sarco/endoplasmic reticulum (SERCA) pump, which refills emptied ER stores [11], [12], [13].

STIM1 and STIM2 are integral type I membrane proteins localized in the ER [13], although a fraction of STIM1 can also be present in the PM [14], [15]. The *N*-terminal parts of both STIMs facing the ER lumen contain of EF-hand-type Ca^2+^-binding domains. STIM2 essentially differs from STIM1 in the *C*-terminal fragment. STIM1 has been identified as an essential controller of SOCE [5], [6], but reports on the role of STIM2 in the SOCE process have been inconsistent. Data show that STIM2 is an inhibitor of SOCE initiated by STIM1 [16], [17]. Other data show that STIM2, in the absence of STIM1, may be responsible for SOCE initiation as a result of even minor changes in the ER [17], [18], [19]. Thus, even in non-excitable cells, the function of STIM proteins has not yet been fully established.

In the case of neuronal cells, in which the main sources of extracellular Ca^2+^ influx are ion channels for neurotransmitter receptors and voltage-dependent Ca^2+^ channels [20], the mechanism of SOCE has only recently become a subject of intensive research [21], [22], [23], [24]. Understanding the mechanism of neuronal SOCE is important with regard to disturbances in calcium homeostasis observed in neurodegenerative diseases (reviewed by [25], [26]). Some studies indeed indicate that SOCE dysfunction, which is accompanied by changes in the expression of Ca^2+^ sensor proteins, may be a cause of some pathologies. For example, in cells with mutated presenilin, which is responsible for early-onset familial Alzheimer's disease, reduced SOCE and impaired STIM2 protein expression were observed [27].

Few studies have addressed the question of the physiological significance of STIM proteins in neurons. Hasan's group showed that ORAI and dSTIM mediated Ca^2+^ influx and Ca^2+^ homeostasis in *Drosophila* neurons [23]. Nieswandt's group questioned the presence of STIM1 in mouse neurons and claimed that STIM2 regulates SOCE in these cells [22]. Using STIM1 or STIM2 knockout mice, they also presented data showing that STIM2 plays a key role in hypoxic neuronal cell death. In contrast, we demonstrated detailed immunolocalization of STIM1 protein in neurons of mouse brain [28]. The STIM1 antibodies we used did not stain neurons in the sections of the STIM1 knockout embryonic brains obtained from Nieswandt's group. The additional characteristic of STIM1 in neurons performed Keil and co-authors [29]. We also showed, for the first time, that puncta-like co-localization of YFP-STIM1 and ORAI1 appeared upon depletion of Ca^2+^ stores in cultured rat neurons [21]. However, the YFP-STIM1(D76A) constitutively active mutant concentrates in puncta even without depletion of neuronal Ca^2+^ stores, and it forces ORAI1 redistribution to those puncta. These observations indicate that STIM1 may play a role in neurons. Recently, one report showed that STIM1 can inhibit L-type voltage-gated Ca^2+^ channels in neurons [24]. Thus, the role of STIM proteins in neurons appears to be more complicated than originally thought.

In the present work, we show that STIM1 and STIM2 play roles in calcium homeostasis in neurons by analyzing both endogenous proteins in nontransfected cells and overexpressed proteins in transfected cells. We report that cultured cortical neurons exhibit SOCE and that STIM1 and STIM2, despite their high sequence similarity and analogous-based domain structures, play distinct roles in this pathway. Based on our data, we postulate that STIM1 is the major SOCE signal transmitter in neurons, whereas STIM2 has primary responsibility for equilibrium calcium homeostasis.

## Results

### *Stim1* and *Stim2* mRNA is present in neurons

**Figure 1:**
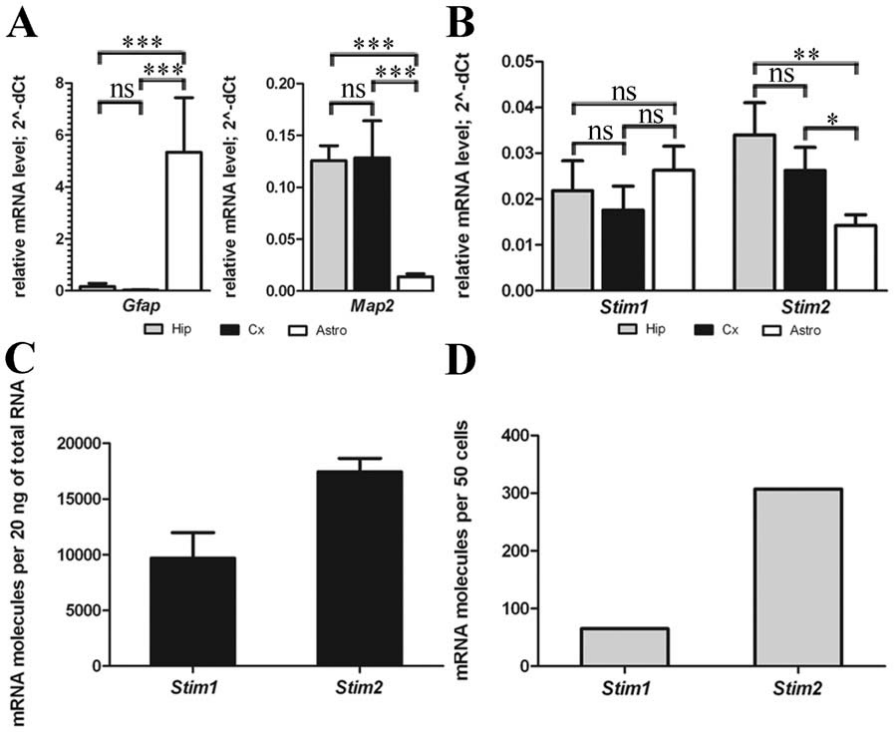
Real-time PCR analysis of *Stim1* and *Stim2* mRNA levels in neurons. mRNA was isolated from primary cultures of neurons and astrocytes. TaqMan primers and probes were used to quantify specific mRNA levels. (**A**) The *Gfap* astrocytic marker and *Map2* neuronal marker are differentially expressed in cultures of cortical neurons (Cx), hippocampal neurons (Hip) and astrocytes (Astro), confirming the purity of the cultures. The expression was related to the *Gapdh* level set as 1 for every culture, using the 2^−dCT^ formula (dCT = CT*_target_* − CT*_Gapdh_*; CT is the cycle threshold). (**B**) *Stim1* and *Stim2* are expressed in neurons of the cortex (Cx) and hippocampus (Hip) and in cortical astrocytes (Astro). The expression was related to *Gapdh* as above. (**C**) The actual number of *Stim1* and *Stim2* mRNA molecules in 20 ng of RNA isolated from cortical neurons was quantified using a standard curve with serial dilutions of cloned *Stim1* and *Stim2*. (**D**) The actual number of *Stim1* and *Stim2* mRNA molecules in laser-dissected hippocampal neurons was quantified as above.

The issue of *Stim1* gene expression in neurons has been controversial [15], [22], [28]. We employed quantitative real-time polymerase chain reaction (PCR) with TaqMan primers and probes to analyze the levels of *Stim1* and *Stim2* expression in primary cultures of cortical and hippocampal neurons and in cortical astrocytes. The purity of the neuronal and astrocytic cultures was verified by analyzing the expression of specific cell type markers: *Map2* for neurons, *Gfap* for astrocytes, and *Aif* for microglia (Figure 1A). High levels of *Map2* and low levels of *Gfap* were detected in neuronal cultures, whereas in astrocytic cultures, the level of *Gfap*, but not *Map2*, was high. *Aif* was not detected in any of the cultures. We report that in astrocytic cultures and both neuronal cultures, similar levels of *Stim1* mRNA were detected. The level of *Stim2* in neurons was approximately two-fold higher than in astrocytes (Figure 1B).

We then calculated the actual number of *Stim1* and *Stim2* mRNA molecules in the analyzed neuronal samples. Quantification was based on the standard curve obtained with serial dilutions of cloned fragments of rat *Stim1* and *Stim2* cDNA. This analysis revealed about 9500 copies of *Stim1* and 17500 copies of *Stim2* mRNA in 20 ng of total RNA extracted from cortical neuronal cultures (Figure 1C). To obtain the exact number of mRNA copies per cell, we used total RNA from laser-dissected hippocampal neurons. We found 65 copies of *Stim1* and 307 copies of *Stim2* mRNA in 50 hippocampal neurons (Figure 1D). Our data demonstrate that both *Stim2* and *Stim1* are expressed in neurons.

### The amount of endogenous STIMs in membrane subfractions from TG-treated neurons is increased

**Figure 2:**
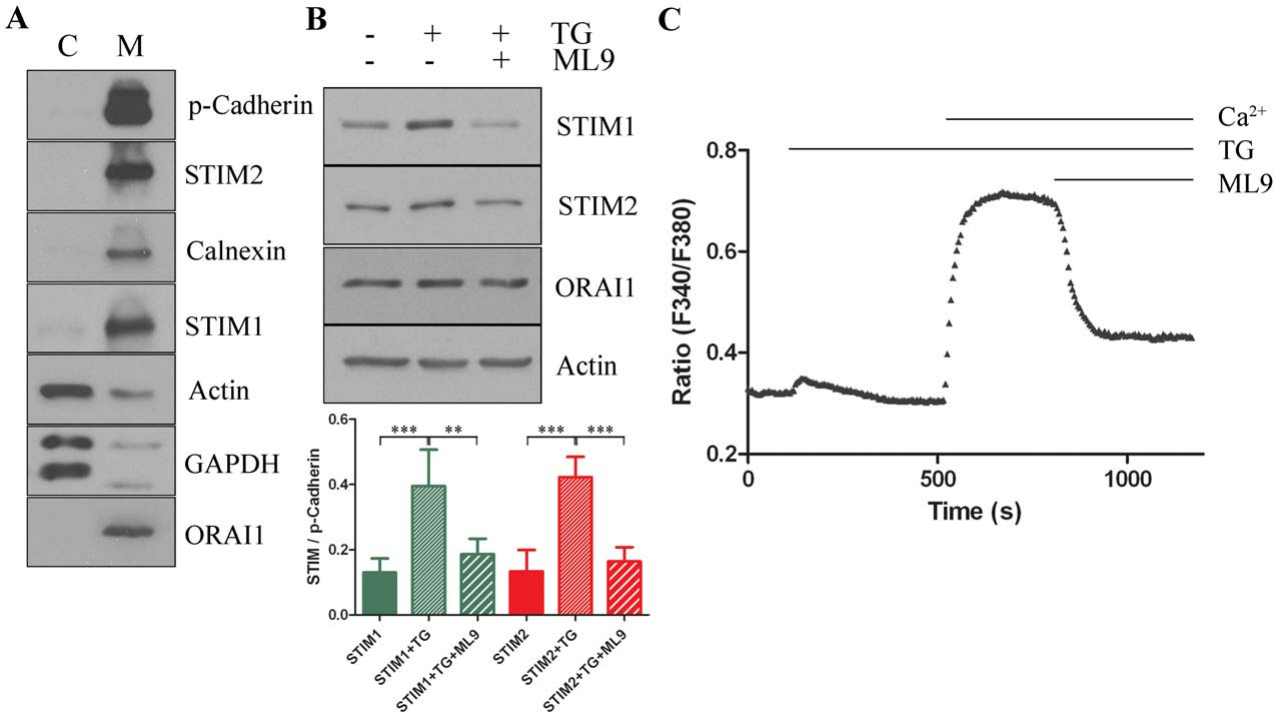
Expression and distribution of endogenous STIM proteins in subcellular neuronal fractions. (**A**) Immunoblots of selected marker proteins demonstrate high separation efficiency for subcellular compartments of neurons on cytosolic (C) and membrane (M) fractions. Proteins were analyzed using the anti-pan Cadherin (plasma membrane), anti-STIM2, anti-Calnexin (ER membrane), anti-STIM1, anti-actin (cytosolic), anti-GAPDH (cytosolic), and anti-ORAI1 antibodies. Notice that STIM and ORAI1 proteins are present only in the membrane fraction. (**B**) Neurons were incubated for 10 min with 2 mM Ca^2+^ (left, −/−), 2 µM TG in 0.5 mM EGTA (middle, +/−), or 2 µM TG in 0.5 mM EGTA followed by the addition of Ca^2+^ in the presence of ML9 for 5 min (right, +/+). The cells were then subjected to fractionation into cytosolic and membrane fractions, and proteins were analyzed by immunoblotting using anti-STIM1, anti-STIM2, anti-ORAI1, anti-p-Cadherin and anti-actin (loading controls) antibodies. Blots were developed using chemiluminescence. Only membrane subfractions are shown. The image shows the results from one representative experiment. Bars indicate mean ± SD from at least three separate experiments. STIM1 or STIM2 bands were normalized to the level of loading control p-Cadherin for each immunoblot. (**C**) Effect of ML9 on SOCE of nontransfected neurons after restoration of extracellular Ca^2+^. ML9 was added 5 min after the readdition of 2 mM CaCl_2_. Raw data are shown. The trace represents the average response of cells measured on a single coverslip from two independent experiments.

Recent studies have reported that STIM proteins function as ER Ca^2+^ sensors in non-excitable cells [5], [6]. To determine whether endogenous STIMs might act as Ca^2+^ sensors also in cultured neurons and whether they can respond to a change in ER Ca^2+^ levels, we examined the effect of TG and the SOCE inhibitor ML9 on their recruitment. We first determined whether the observations with the presence of *Stim1* and *Stim2* mRNA in neurons can be confirmed by studying endogenous proteins expressed in cultured neurons. To achieve this goal, we isolated membrane and cytosolic fractions from neurons cultured under various calcium conditions, separated them by sodium-dodecyl sulfate-polyacrylamide gel electrophoresis (SDS-PAGE), and performed immunoblotting (Figure 2A). The blots containing cytosolic and membrane proteins from untreated cortical neurons were analyzed for the presence of markers with molecular weight characteristics for selected proteins, such as p-Cadherin (PM marker), calnexin (ER marker), actin, GAPDH (cytosolic markers), STIM2, STIM1, and ORAI1. The immunoblots showed that the cytosolic protein fraction was devoid of PM and ER membranes, whereas the membrane protein fraction contained both p-Cadherin and calnexin and a small amount of actin and GAPDH. Figure 2A shows that ORAI1 and both STIMs are present in membranes from cortical neurons and absent from the cytosolic fraction. During subsequent experiments the cytosolic and membrane fractions were isolated from neurons cultured in Ca^2+^-containing medium (−/−), from neurons that were transferred to low EGTA-containing medium and treated with thapsigargin (TG; +/−), and from neurons to which the SOCE inhibitor ML9 was added for 5 min after incubation with TG (+/+). The blots were immunostained with STIM1, STIM2, ORAI1, p-Cadherin and actin antibodies and developed by chemiluminescence (Figure 2B). The immunoblotting data revealed an increased amount of endogenous STIMs in membranes from TG-treated neurons. ML9 inhibited such an increase in a slightly greater extent for STIM2 (*p*<0.001) than for STIM1 (*p*<0.01) (Figure 2B). The stable level of ORAI1 in the membrane fractions confirms the specificity of redistribution of endogenous STIMs to the PM. To confirm the inhibitory activity of ML9, the intracellular level of Ca^2+^ ions was measured using a Fura-2 indicator (Figure 2C). In cortical neurons incubated in EGTA-containing medium, TG treatment followed by transfer to a medium containing CaCl_2_ in the millimolar range induced Ca^2+^ influx via SOC channels. The subsequent incubation of cells in ML9 inhibited SOCE by approximately 70%. Thus, the data show that the intracellular distribution of both STIM1 and STIM2 in nontransfected neurons is sensitive to the level of Ca^2+^ in the ER and the SOCE inhibitor (Figure 2), demonstrating that they have the ability to function as SOCE sensors.

### YFP-STIM1 and YFP-STIM2 form puncta with ORAI1 in response to TG or Ca^2+^ chelator

**Figure 3:**
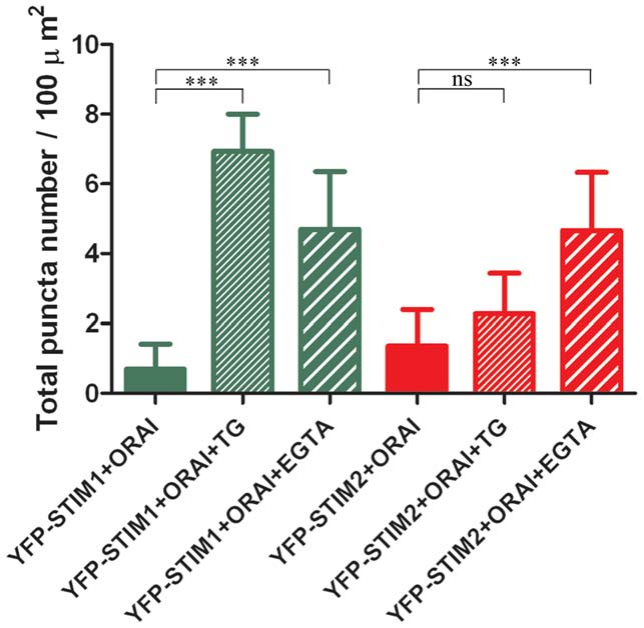
Puncta quantification of YFP-STIM1 or YFP-STIM2 co-expressed with ORAI1. Number of STIMs-ORAI1 puncta calculated per µm^2^ of PM area in neurons. Data were obtained from images of neurons co-expressing ORAI1 and YFP-STIM1 (green bars) or YFP-STIM2 (red bars) in the presence of 2 mM extracellular Ca^2+^ before store depletion, 10 min following treatment with 2 µM TG in 0.5 mM EGTA or 2 mM EGTA alone. The confocal images were analyzed using ImageJ software. Data are expressed as the average of at least 25 cells for each of the transfection conditions. ***p<0.001, compared with control (cells maintained in 2 mM Ca^2+^); ns, not significant (ANOVA). Bars indicate mean ± SD from at least three separate experiments.

To better understand the role of STIM proteins, we compared their ability to form puncta in cultured cortical neurons co-transfected with plasmids encoding either YFP-STIM1 and ORAI1 or YFP-STIM2 and ORAI1. This allowed us to directly correlate intracellular Ca^2+^ levels with the number of STIMs and ORAI1 complexes. Twenty-four hours after transfection, cells were transferred to a medium with low EGTA and treated with TG to deplete Ca^2+^ stores. This induced a change in fluorescence distribution in the cells from dispersed in control cells incubated with 2 mM Ca^2+^ (Figure S1, upper panels) to aggregated after TG treatment (Figure S1, middle panels). We developed a method for puncta counting and found that depletion of Ca^2+^ from ER stores by the presence of TG increased the number of STIM1-ORAI1 puncta more than nine-fold, whereas the number of STIM2-ORAI1 puncta increased less than two-fold, which was not statistically significant (Figure 3). This indicated, that STIM1 is a major sensor of ER Ca^2+^ levels during SOCE. The function of STIM1 in neurons being different from STIM2 was also concluded when the yellow fluorescent protein (YFP) fluorescence of STIM protein redistribution was analyzed in transfected neurons transferred to a medium with high EGTA without TG (Figure S1, bottom panels). In YFP-STIM1 cells, the number of puncta increased approximately six-fold, which was slightly less than in the presence of TG-containing medium but still statistically significant (*p*<0.001). In YFP-STIM2 cells, the number of puncta under identical conditions significantly increased four-fold (*p*<0.001) (Figure 3). This was a two-fold increase compared with the number observed in the presence of TG, indicating that under high EGTA conditions, STIM2 became activated and STIM1 became inhibited. These observations suggest that STIM1 is likely involved in TG-induced SOCE, and STIM2 is mostly active after EGTA-driven depletion of extracellular Ca^2+^. The latter leads to calcium homeostasis breakdown and the induction of constitutive calcium entry mechanism to compensate for Ca^2+^ leakage into the extracellular space.

### Co-expression of YFP-STIM1 with ORAI1 activates SOCE after TG-induced ER depletion

**Figure 4:**
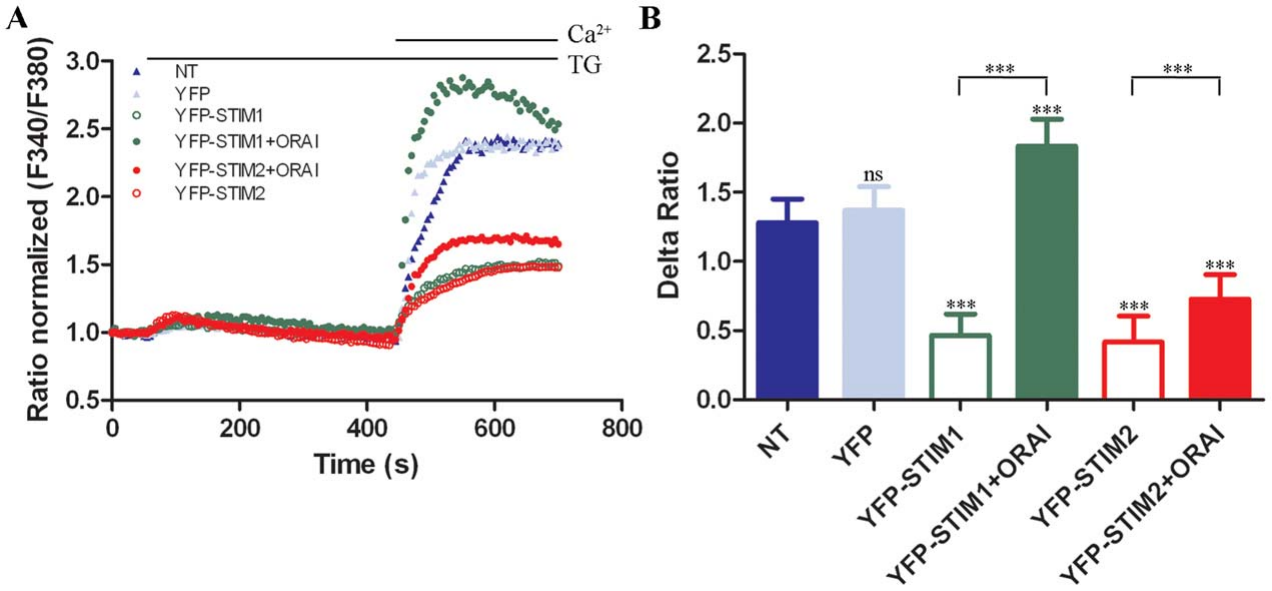
Analysis of SOCE in transfected cortical neurons in response to store depletion by TG. (**A**) Averaged traces from 15–20 cells per trace from at least three experiments of intracellular Ca^2+^ (F_340_/F_380_) levels obtained by ratiometric Fura-2 analysis of neurons overexpressing YFP-STIM1 ± ORAI1, YFP-STIM2 ± ORAI1, YFP, or nontransfected (NT). Measurements were started in a buffer supplemented with 0.5 mM EGTA, which was then replaced by a buffer with 0.5 mM EGTA and 2 µM TG. Finally, 2 mM CaCl_2_ was added to the medium to detect Ca^2+^ ion entry. F_340_/F_380_ values beginning just before the addition of TG were normalized to the same values (1). (**B**) Summary data showing the maximum TG-induced Ca^2+^ ion influx in neurons described in (**A**). Data are expressed as the Delta Ratio (± SD), which was calculated as the difference between the peak F_340_/F_380_ ratio after extracellular Ca^2+^ was added and its level immediately before the addition of Ca^2+^. ***p<0.001, compared with control (NT); ns, not significant (ANOVA).

The differential participation of STIM1 and STIM2 in puncta formation led us to determine their effects on intracellular Ca^2+^ levels. Cortical neurons were loaded with the Fura-2 Ca^2+^ indicator in 2 mM CaCl_2_-containing medium, washed, and transferred to a 0.5 mM EGTA medium to initiate measurements. TG was then added. After 390 s, the medium was changed to 2 mM CaCl_2_ with continuous detection of Fura-2 signals. The peaks appearing after the exchange of medium were proportional to the number of open SOC channels (Figure 4A). The data in Figure 4B are expressed as the Delta Ratio, which is defined here as the difference between the F_340_/F_380_ ratio at the peak obtained as a result of the extracellular Ca^2+^ addition and the average F_340_/F_380_ ratio detected just before the Ca^2+^ addition. The Delta Ratio values in these experiments were approximately 1.3 for nontransfected cells (closed triangles) and approximately 1.4 for cells transfected with empty YFP vector (open triangles). This difference was not statistically significant (*p*>0.5) (Figure 4B). Thus, the transfection procedure itself and YFP expression did not have significant effects on the membrane state or on the level of SOCE. Surprisingly, the transfection of neurons with either YFP-STIM1 or YFP-STIM2 alone led to inhibition of SOCE to a Delta Ratio value of approximately 0.5 (Figure 4A, green and red open circles, respectively), and this effect was statistically significant (Figure 4B). However, co-expression with ORAI1 (green closed circles) enhanced SOCE approximately four-fold compared with cells transfected with YFP-STIM1, which was well above the level observed in control cells (triangles). Co-expression of ORAI1 in cells with YFP-STIM2 enhanced SOCE less than two-fold compared with cells transfected only with YFP-STIM2, which was still below the level observed for control cells (Figure 4B). These data show a good correlation between the number of puncta observed in Figure 3 and the extent of SOCE (Figure 4) and indicate that STIM1 plays a major role in SOCE after TG-induced ER depletion in neurons.

### SOCE inhibitors differentiate Ca^2+^ influx in TG-treated transfected neurons with either STIM1 or STIM2

**Figure 5:**
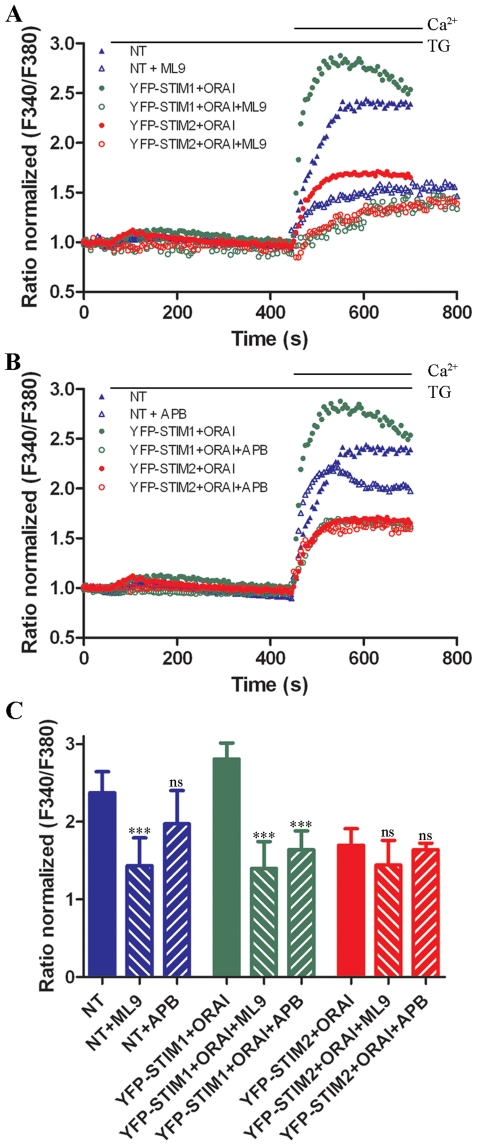
Effect of SOCE inhibitors ML9 and 2-APB on TG-sensitive SOCE in transfected cortical neurons. (**A–B**) Cortical neurons were co-transfected with YFP-STIM1 and ORAI1 or YFP-STIM2 and ORAI1 or nontransfected (NT). Experiments were performed as described in Figure 4, but (**A**) 100 µM ML9 or (**B**) 50 µM 2-APB was added before store depletion with TG in the presence of inhibitors. Ca^2+^ (2 mM) was then added to assess SOCE. Each trace represents the average response of cells measured on a single coverslip from three independent experiments. F_340_/F_380_ values beginning just before the addition of TG were normalized to the same values (1). (**C**) The average increase in Ca^2+^ entry observed after the addition of 2 mM CaCl_2_ to transfected or nontransfected cells in the absence or presence of the SOCE inhibitors from experiments performed as described in (**A–B**). The data show the peak of the F_340_/F_380_ ratio after extracellular Ca^2+^ was added. ***p<0.001; ns, not significant (ANOVA). Error bars represent the standard error from three independent experiments.

To further explore the difference between the modes of action of the STIM proteins, we analyzed the level of SOCE in the presence of ML9 (Figure 5A) or 2-APB, another SOCE inhibitor (Figure 5B). ML9 or 2-APB was added before TG treatment to the cells incubated in low EGTA-containing medium, and their concentrations were maintained during the experiments. The effect of ML9 on TG-evoked SOCE was highly effective in nontransfected neurons and significantly lowered its maximum (Figure 5A and C). 2-APB, however, had little effect in nontransfected control cells (Figure 5B and C). In transfected cells, the inhibitory effect of SOCE inhibitors depended on the type of overexpressed STIM protein. ML9 treatment reduced the strength of SOCE two-fold in cells co-transfected with YFP-STIM1 and ORAI1 (*p*<0.001) but had no significant effect on cells expressing YFP-STIM2 and ORAI1 (Figure 5A and C). 2-APB had the same effect on co-transfected cells, inhibiting cells with STIM1 but not with STIM2 (Figure 5B and C). Thus, the effect of these drugs can be differentiated if Ca^2+^ influx is induced by STIM1 or STIM2. The data confirm that STIM1 protein is involved in TG-induced SOCE because its Ca^2+^ influx is sensitive to both SOCE inhibitors. However, STIM2 protein appears to be less important because its involvement in Ca^2+^ influx is insensitive to these inhibitors.

### Spontaneous store repletion occurs to a higher extent with YFP-STIM2 cells than YFP-STIM1 cells

**Figure 6:**
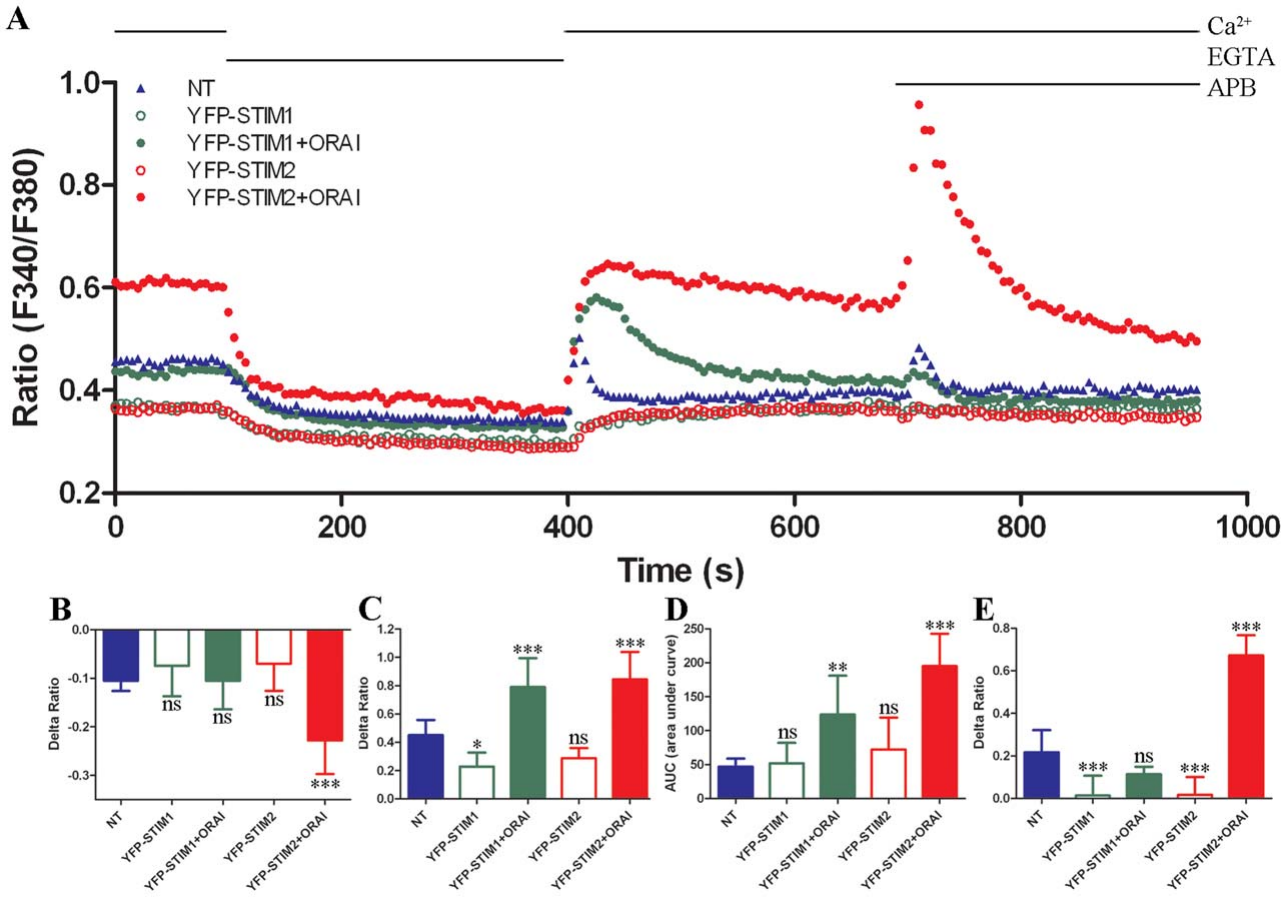
Analysis of spontaneous store repletion in transfected neurons. (**A**) Cytosolic Ca^2+^ measurements were performed in cells overexpressing YFP-STIM1 ± ORAI1 or YFP-STIM2 ± ORAI1 or in nontransfected (NT) cells. The experiments started in the presence of 2 mM CaCl_2_, followed by transfer to Ca^2+^-free medium. A buffer was then supplemented with 2 mM CaCl_2_ for 5 min to monitor intracellular Ca^2+^ restoration. Finally, 50 µM 2-APB was added. Raw (not normalized) traces are shown, which represent the average of at least 30–50 cells from two independent experiments. (**B**) Summary of basal Ca^2+^ at the beginning of the experiment. Data are shown as the Delta Ratio values, which were calculated as the difference between the F_340_/F_380_ ratio after extracellular Ca^2+^ was removed and its maximum level at the beginning of the experiment. (**C–D**) Analysis of constitutive calcium entry in transfected cortical neurons. Traces were normalized to 1 and are shown as a Supplementary Material (Figure S2A). (**C**) The average increases in Ca^2+^ entry (peak Ca^2+^ rise) observed after the addition of 2 mM CaCl_2_ to transfected cells compared with nontransfected cells are shown. (**D**) Statistical evaluation of the integrated Ca^2+^ responses (area under the curve [AUC]) is shown. (**E**) Effect of 2-APB on Ca^2+^ responses (peak Ca^2+^ rise). Traces normalized to 1 are shown as Supplementary Material (Figure S2B). The average Ca^2+^ responses to 50 µM 2-APB in 2 mM Ca^2+^ in cells transfected or nontransfected are shown. (**B–E**) *p<0.05, ***p<0.001, compared with control; ns, not significant (ANOVA). Bars indicate mean ± SD from at least 30–50 cells measured in two independent experiments.

The average cytoplasmic resting Ca^2+^ level depends on the set of proteins with which the cells were transfected (Figure 6A). The cells expressing either YFP-STIM1 or YFP-STIM2 alone (open green and open red circles, respectively) had lower resting Ca^2+^ levels than nontransfected cells (blue triangles), whereas cells co-expressing YFP-STIM2/ORAI1 had a much higher level of resting Ca^2+^ (closed red circles *vs*. blue triangles). The difference was statistically significant (*p*<0.001). In contrast, YFP-STIM1/ORAI1 transfectants showed similar resting levels without any significant difference compared with nontransfected cells (Figure 6A, closed green circles *vs*. blue triangles). Subsequent incubation of cells in a high EGTA-containing medium led to a slow decrease in intracellular free Ca^2+^ ions to similar levels in all analyzed cell types. The highest decrease (and the only one that was statistically significant, *p*<0.001) was observed in neurons co-transfected with YFP-STIM2/ORAI1 (Figure 6A and B) because those cells had the highest resting level of free Ca^2+^ before the medium exchange. The exchange of the medium to the one with the original Ca^2+^ concentration induced constitutive calcium entry in nontransfected cells as well as in double transfectants (YFP-STIM1/ORAI1 and YFP-STIM2/ORAI1) (Figure 6A and C), after which cytoplasmic free Ca^2+^ levels returned to resting levels characteristic of cells expressing particular proteins (Figure 6A). Cells transfected with STIM1 or STIM2 alone did not develop the peak Ca^2+^ signal, but their free cytoplasmic Ca^2+^ levels slowly increased back to the original levels (Figure 6A). The peak observed in nontransfected cells was much shorter and lower than in both double transfectants. The peaks of the Ca^2+^ response were quite similar in YFP-STIM1/ORAI1 and YFP-STIM2/ORAI1 neurons. However, the subsequent plateau phase, which reflects integrated Ca^2+^ influx, was much higher in YFP-STIM2/ORAI1 neurons than in YFP-STIM1/ORAI1 neurons (Figure 6A and D). The above data confirm that STIM1 and STIM2 differentially participate in the regulation of resting Ca^2+^ levels and show that they differentially contribute to the replenishment of Ca^2+^ stores. An additional argument for their distinct roles came from the experiments in which 50 µM 2-APB was used (Figure 6A and E). In nontransfected and both double-transfected cells, the sole treatment with 2-APB evoked a Ca^2+^ signal. Although the response of nontransfected cells and YFP-STIM1/ORAI1 transfectants to 2-ABP was rather weak and not significant, YFP-STIM2/ORAI1 transfectants developed well-pronounced Ca^2+^ transient after 2-APB treatment. This is interesting when comparing these results with the earlier observations that TG-induced SOCE in those transfectants was insensitive to 2-APB (Figure 5B and C). Figure 6 shows that a major role of STIM2 in neurons is the control of ER resting Ca^2+^ levels and their maintenance by tuning Ca^2+^ entry. STIM1 may also regulate constitutive calcium entry.

## Discussion

In the present study, we examined the functions of STIM1 and STIM2 in the process of SOCE. We found that under conditions of ORAI1 overexpression in cortical neurons, STIM1 is the main activator of TG-induced SOCE, whereas STIM2 regulates basal luminal Ca^2+^ levels and activates the constitutive calcium entry with the additional involvement of STIM1. Our findings solve some of the crucial issues related to the mechanism of SOCE in neurons.

Berna-Erro et al., using reverse transcription PCR performed on material isolated by microscopy laser capture from hippocampal neurons, found *Stim2* bands on electrophoretic gels, but no band for *Stim1* was detected [22]. The authors claimed that *Stim1* is expressed in other cells than in neurons. The present study showed that cortical and hippocampal neurons express both *Stim* mRNA using real-time PCR and specific TaqMan primers and probes. We also used cDNA from 50 laser-captured neurons. The difference between our data and the results of Berna-Erro et al. can be explained by the different PCR protocols and methods of product identification. They analyzed the products of PCR on electrophoretic gels. Unclear is with which cycle the band of *Stim2* was obtained and whether the PCR reaction can be considered quantitative. We monitored the PCR reaction during the exponential phase and also applied absolute quantification to calculate the exact copy number of *Stim1* and *Stim2* molecules per 50 cells for laser-captured neurons or 20 ng for scraped material from cultures. According to our data, the levels of *Stim1* expression in neurons and astrocytes are similar.

We also showed that Ca^2+^ store depletion induces an increase in the association of STIM1 and STIM2 with the membrane fraction. Taking into account that STIMs have been found only in the membranes, one of the possibilities is that ER vesicles are pelleted with the increasing efficiency as a result of STIM aggregation and its interaction with ORAI present in plasma membrane. Another explanation for this result could be that the recovery of ER membrane is altered under the conditions of TG stimulation. However, this seems unlikely since the level of calnexin, ER membrane marker, did no change as a result of TG treatment (not shown). In turn, the addition of 100 µM ML9 reduced the level of both STIMs in the membrane fraction and inhibited endogenous SOCE. These results are consistent with data obtained from non-excitable cells by Smyth et al., in which the rearrangements of STIM proteins was a reversible process, and the inhibition of SOCE by ML9 was attributable to the reversal of STIM localization [11].

We observed a strong STIM1-dependent signal and the dependence of SOCE on its inhibitors, similar to other cell types. Both inhibitors (ML9 and 2-APB) are widely accepted as affecting SOCE, although their precise mechanisms of action are unclear. ML9 was assumed to regulate SOCE by means of cytoskeleton modulation, but Putney's group showed that ML9 may block the activity of other kinases to inhibit SOCE [11]. Nevertheless, ML9 and 2-APB strongly inhibited the signal in YFP-STIM1/ORAI1 and endogenous STIM-ORAI1 in nontransfected neurons, demonstrating that SOCE occurred.

From the discovery of the role of STIM proteins in store-operated Ca^2+^ signal formation [5], [6], STIM1 protein is clearly a key player in SOCE. The recent report showed that STIM1 can inhibit L-type voltage-gated Ca^2+^ channels in neurons [24], indicating that the mechanisms of calcium homeostasis in neurons are more complex than anticipated, and additional links might exist between voltage-dependent Ca^2+^ channels and SOCE. We propose that STIM1 acts as a controller of Ca^2+^ leakage. Our results show that after incubation of neurons with high EGTA-supplemented buffer, constitutive calcium entry was increased in YFP-STIM1/ORAI1 neurons. Thus, it is not surprising that we observe under such conditions an increase in the number of YFP-STIM1 and ORAI1 complexes, similarly as in case of YFP-STIM2 and ORAI1 puncta. In non-excitable HEK293 cells STIM1 and ORAI1 co-expression had only very modest effects on Ca^2+^ leak [30], [31]. Moreover, puncta of STIM1 were shown to be formed in HeLa cells, however much less rapidly than STIM2 puncta, when ER Ca^2+^ stores were slowly depleted by EGTA treatment [18]. Also, it has been suggested that the STIM1 can create two types of puncta: “resting” oligomers in cells with replenished Ca^2+^ stores or higher-order oligomers in store-depleted cells [32]. Thus, it seems that STIM1 might response to EGTA treatment in non-excitable cells, but with a very modest sensitivity. It is therefore possible that in rat cortical neurons it has higher sensitivity to EGTA.

The role of STIM2 protein was much more elusive and difficult to interpret. Our results show the relative inability of STIM2 protein to form puncta after complete store depletion, which is believed to be necessary for SOCE to occur [5]. However, overexpressed STIM2 can build into STIM1 puncta [16], acting as an inhibitor of SOCE, which strongly suggests completely different modes of action of this protein. On the other hand, the inhibitory effect of STIM2 on SOCE may depend on the construct concentration. Putney's group demonstrated that STIM2 overexpression at a lower plasmid concentration did not inhibit TG-activated Ca^2+^ entry and thus no longer behaved like a partial agonist [17]. Nonetheless, we did not observe statistically significant inhibitory influence of ML-9 or 2-APB on SOCE activated by complete store depletion in transfected cells. However, from the results shown in nontransfected cells, it is difficult to exclude the scenario in which STIM2 also contributes to endogenous SOCE in neurons. Especially that in Figure 2B we show that ML9 inhibits STIM2 enrichment in membranes after TG treatment.

Another possible role of STIM2 protein is the regulation of resting ER Ca^2+^ level [18], [30]. In the resting cell, the ER Ca^2+^ load is in equilibrium with cytoplasmic free Ca^2+^ levels, and STIM2 also influences cytoplasmic Ca^2+^ load. Indeed, we observed a strong increase in resting cytoplasmic Ca^2+^ levels in YFP-STIM2/ORAI1 co-transfectants. This result may reflect the YFP or overexpression-induced aggregation of YFP-STIM2 and ORAI1 constructs. However, the elevated baseline we observe only in the case of YFP-STIM2 + ORAI 1 but not of YFP-STIM1 + ORAI1 transfectants, what makes this possibility implausible. Another important finding is that YFP-STIM2/ORAI1 does not inhibit basal Ca^2+^ entry, as in case of SOCE induced by TG but, contrariwise, largely increase the Ca^2+^ level, suggesting that STIM2 protein significantly interferes with ORAI1 but only with a slight decrease in Ca^2+^ level in the ER. As previously reported [18], STIM2 has lower ER Ca^2+^ sensitivity than STIM1 and therefore STIM2 translocates to puncta with ORAI1 and activates constitutive calcium influx upon smaller decreases in ER Ca^2+^ concentration compared with STIM1. Extended incubation in the EGTA-containing medium leads to a decrease in cytoplasmic Ca^2+^ levels and presumably also slowly empties ER Ca^2+^ stores. The hypothesis of STIM2 as an equilibrium regulator is strongly supported by kinetic studies of STIM oligomerization performed by Ikura's group [33], who showed approximately 70-fold slower aggregation of STIM2 compared with STIM1. This result is further corroborated by the effect described for 2-APB on resting cells. The burst of Ca^2+^ into the cytoplasm of STIM2-ORAI1 cells after STIM-independent ORAI1 induction attributable to 2-APB binding to the channel was described by Putney's group [34]. Thus, this poorly characterized Ca^2+^ channel inhibitor, which is believed to be a SOCE blocker, was able to compensate for the increase in resting Ca^2+^ level evoked by STIM2-ORAI1 co-transfection. Notably, our results showing a stimulatory effect of 50 µM 2-APB on cytosolic Ca^2+^ levels in STIM2/ORAI1-expressing neurons are consistent with published studies demonstrating a similar effect of 2-APB on cytosolic Ca^2+^ levels [35], [36] and on I_CRAC_ (Ca^2+^ release activated current) [19] in non-excitatory cells. However, this effect is better known than understood.

Some researchers believe, however, that at least in some cell types, such as T-lymphocytes or neurons, STIM2 is responsible for SOCE. Rao's group [37] showed that STIM2 was able to reconstitute SOCE in STIM1-deficient T-lymphocyte cells, even if STIM1 was the main SOCE regulator in wildtype T-lymphocytes. Nieswandt's group claimed that STIM2, but not STIM1, regulates SOCE in neurons [22]. Overall, we report that cultured cortical neurons exhibit SOCE and that STIM1 and STIM2 play distinct roles in this process. Our results indicate that STIM1 is the main SOCE signal transmitter in neurons, whereas STIM2 is responsible for calcium homeostasis clearly influencing resting luminal Ca^2+^ levels and activating the constitutive calcium entry. Surprisingly, STIM1 also had an effect on basal Ca^2+^ leakage. In summary, our data show that machinery of SOCE works in neurons as in non-excitatory cells. However, it still remains unclear if SOCE plays a role of direct neurotransmission modulator or is responsible for indirect neuronal function regulator, what was suggested by Tsim and co-workers [38], [39].

## Materials and Methods

### Primary neuron cultures

The cortical neuron cultures were prepared from 19-day-old embryonic (E19) Wistar rat brains. Animal care was in accordance with the European Communities Council Directive (86/609/EEC). The experimental procedures were approved by the Local Commission for the Ethics of Animal Experimentation no. 1 in Warsaw. Brains were removed from rat embryos and collected in cold Hanks solution supplemented with 15 mM HEPES buffer and penicillin/streptomycin. The cortex was isolated, rinsed three times in cold Hanks solution, and treated with trypsin for 20 min. Tissue was then rinsed in warm Hanks solution and dissociated by pipetting. Primary cortical neurons were plated at a density of either 15×10^4^ per 13 mm glass coverslip or 30×10^4^ per 19 mm glass coverslip coated with laminin (2 µg/ml, Roche) and poly-D-lysine (2 µg/ml, Sigma). Cells cultured in 24-well plates were used for immunofluorescence measurements, whereas cultures on 12-well plates were used for measurements of intracellular Ca^2+^ accumulation. For immunoblotting, neurons were seeded on poly-D-lysine-coated Petri dishes at 7×10^6^ cells/plate. Neurons were grown in Neurobasal medium (Invitrogen) supplemented with B27 (Invitrogen), 0.5 mM glutamine (Sigma), 12.5 µM glutamate (Sigma), and a penicillin (100 U/ml)/streptomycin (100 µg/ml) mixture (Gibco). Cultures were maintained at 37°C in humidified 5% CO_2_/95% air. The experiments were performed on 8- to 10-day-old cultures.

### Real-time PCR analysis and laser capture microscopy

Scraped material from neuronal and astrocytic cultures (isolated according to [40]) were lysed in RNA Lysis Buffer RLT. Total RNA was then isolated with an RNeasy Plus Kit (Qiagen). Total RNA from 50 laser-captured neurons was isolated with an RNeasy Micro Kit (Qiagen). The laser capture was performed by Zeiss (http://www.zeiss.de/microdissection). cDNA was synthesized with random hexamer primers and SuperScript III RNase H-Reverse Transcriptase (Invitrogen). The cDNA of 50 dissected cells was preamplified with the TaqMan PreAmp Master Mix Kit (Applied Biosystems) during 10 cycles. Our experimental data showed approximately 250-fold amplification.

The samples were examined by real-time PCR in a 7900HT Real Time PCR System (Applied Biosystems). Commercial TaqMan primers and probes (Applied Biosystems) were used to quantify specific mRNA levels: *Gapdh* (control), *Map2* (neuronal marker), *Gfap* (astrocytic marker), *Stim1*, and *Stim2*. For relative quantification, the results were related to the *Gapdh* mRNA level set as 1, assuming equal amplification efficiency for each primer pair and using the 2^−dCT^ formula (dCT = CT*_target_* − CT*_Gapdh_*; CT is the cycle threshold). For absolute quantification, we used a standard curve with serial dilutions of linearized plasmid with cloned fragments of rat *Stim1* and *Stim2* cDNA (10^6^-10^1^ molecules). To calculate exact mRNA copy numbers per neuron, we ran 1/250 of the preamplified material, corresponding to the initial material obtained from 50 cells.

### Transfections

A full-length ORAI1 cDNA vector was purchased from Open Biosystems in the pCMV-SPORT6 vector. YFP-STIM1 and YFP-STIM2 constructs were a generous gift from Dr. Tobias Meyer, Stanford University. Cortical neurons grown on 13 mm or 19 mm coverslips were transiently transfected with the aid of Lipofectamine 2000 Reagent (2 µl or 4 µl per well in 24- or 12-well plates, respectively; Invitrogen) according to the manufacturer's protocol. We used ORAI1 cDNA and one of the YFP-STIM constructs (STIM1 or STIM2) at 0.4 µg/0.8 µg of DNA per well for each construct, respectively. As a control, 0.8 µg of YFP cDNA per well was used. The cells were exposed to the mixture of plasmid DNA and Lipofectamine 2000 for 3.5 h in a serum-free culture medium. Afterward, the neurons were returned to saved conditioned Neurobasal supplemented with B27, 0.5 mM glutamine, 12.5 µM glutamate, and penicillin/streptomycin. The experimental treatments were initiated 24 h after transfection.

### Subcellular fractionation and immunoblotting

For immunoblotting, cortical neurons stimulated for 10 min with 2 mM CaCl_2_, 2 µM TG in 0.5 mM EGTA, or 2 µM TG in 0.5 mM EGTA followed by 5 min incubation with 100 µM ML-9 were subjected to subcellular fractionations. The cell pellets (∼2×10^6^ cells) were suspended in ice-cold buffer A (250 mM saccharose, 10 mM NaCl, 1.5 mM MgCl_2_, 0.5 mM DTT, 0.1 mM PMSF, complete EDTA-free protease inhibitors cocktail [Roche] in 10 mM Tris-HCl, pH 7.5) for 30 min and lysed with an insulin syringe (18×). Nuclei were removed by centrifugation at 1,000×*g* for 10 min at 4°C. The supernatants were subjected to centrifugation at 8,000×*g* for 10 min to remove the pellets corresponding to the mitochondria fractions. The resulting supernatants, containing cytosolic and membrane proteins, were mixed with 0.11 vol of buffer B (1.4 M NaCl, 30 mM MgCl_2_, 300 mM Tris-HCl, pH 7.5) and ultracentrifuged at 100,000×*g* for 1 h at 4°C. The obtained supernatants represented the cytosolic fractions. The membrane proteins (pellets) were extracted with buffer C (15% glycerol, 0.4 M NaCl, 1 mM DTT, 0.1% NP-40, 0.5% Triton, 1 mM PMSF, 20 mM Tris-HCl, pH 7.8 with protease inhibitor mixture), and both fractions were stored at −80°C.

Membrane and cytosolic protein extracts were resolved by 10% SDS-PAGE, transferred to a Protran nitrocellulose membrane (Whatman), and blocked for 2 h at room temperature in TBST (50 mM Tris-HCl [pH 7.5], 150 mM NaCl, 0.1% Tween 20) plus 5% dry non-fat milk. Nitrocellulose sheets were then incubated in blocking solution with primary antibodies against STIM1 (ProteinTech Group), STIM2 (Alomone Laboratories), pan- Cadherin (Abcam), calnexin (Sigma), actin (Sigma), GAPDH (Santa Cruz Biotechnology), and ORAI1 (Cell Signaling) at 4°C overnight. The appropriate horseradish peroxidase-conjugated secondary antibody IgG (Sigma) was added at a dilution of 1∶25,000 for 1 h. The peroxidase was detected with enhanced chemiluminescence detection (Amersham Biosciences). The intensity of the bands was measured using a GS-800 Calibrated Densitometer and Quantity One software (Bio-Rad).

### Single-cell Ca^2+^ measurements

Single-cell Ca^2+^ levels in cortical neurons were recorded using the ratiometric Ca^2+^ indicator dye Fura-2AM [41], and imaging was performed as described previously [27]. Cells grown on coverslips were loaded with 2 µM acetoxymethyl (AM) ester of Fura-2 for 30 min at 37°C in a solution containing 145 mM NaCl, 5 mM KCl, 0.75 mM Na_2_HPO_4_, 10 mM glucose, 10 mM HEPES (pH 7.4), 1 mM MgCl_2_, and 0.1% bovine serum albumin (BSA, standard buffer) supplemented with 2 mM CaCl_2_ at 37°C. After rinsing, coverslips were transferred on a thermostatic chamber that was placed on the stage of a Nikon Diaphot inverted epifluorescence microscope equipped with a fluo×40/1.3 NA oil immersion objective lens. The Ludl Lep MAC 5000 filter wheel system loaded with a Chroma Fura-2 filter set was used for illumination of specimens. Images were acquired using a Andor Luca^EM^R EMCCD digital camera (Andor Technology). Intracellular Ca^2+^ levels in individual neuronal cell bodies were calculated after subtracting background fluorescence by measuring the ratio of the two emission intensities for excitation at 340 and 380 nm (F_340_/F_380_). The ratio of emissions at 510 nm was recorded every 5 s. Ca^2+^-free solution instead of CaCl_2_ contained 0.5 mM EGTA. Data processing was performed using Andor iQ (Andor Technology) and Microsoft Excel software.

### Immunocytochemistry

For the immunocytochemical experiment, transfected neurons cultured on coverslips and stimulated for 10 min with 2 mM CaCl_2_, 2 µM TG in 0.5 mM EGTA, or 2 mM EGTA alone were fixed in ice-cold 4% paraformaldehyde (PFA) and 4% sucrose in phosphate-buffered saline (PBS) for 15 min at room temperature. After permeabilization in 0.1% Triton X-100 and blockade with 5% BSA in PBS for 30 min, an antibody against ORAI1 diluted in blocking solution (10% horse serum, 5% sucrose, 2% BSA, and 0.1% Triton X-100 in PBS, pH 7.5) was applied for 2 h at room temperature. The ORAI1 antibody (rabbit) was custom-made against a 15 amino acid peptide (27–41 amino acids) from near the *N*-terminus of human ORAI1 and affinity-purified (Invitrogen). The staining was detected using anti-rabbit Alexa Fluor 594-conjugated secondary antibody (Molecular Probes) in blocking solution for 45 min at room temperature. To visualize the nuclei of transfected cells, we included the Hoechst 33258 dye (Invitrogen) in the wash for 5 min after the secondary antibody incubation. Coverslips were mounted on slides with Mowiol (5% polyvinyl alcohol [FLUKA], 12% glycerin, 50 mM Tris-HCl, pH 8.5). Images of immunofluorescence were acquired using a TCSSP2 Leica confocal microscope with LCS software.

### Puncta analysis

The number of YFP-STIM1/ORAI1 and YFP-STIM2/ORAI1 puncta within neurons treated with 2 mM CaCl_2_, 2 µM TG with 0.5 mM EGTA, or 2 mM EGTA alone was determined using NIH ImageJ software. For the analysis, single 0.25 µM thick confocal scans with the largest-diameter cell bodies from the “green” and “red” channels (Figure S1) were applied. The dataset was then deconvoluted, resulting in an improved signal-to-noise ratio and resolution. After deconvolution, a background subtraction was performed followed by the colocalization analysis compiled from both channels. The final processed images were thresholded and masked around the cell periphery. Individual regions corresponding to integrated STIM and ORAI1 puncta were calculated from the masked images by using the particle analysis function. The same setting (size: 0.32–1.62 µm^2^; circularity: 0–1) was used for all images. Thus, only puncta localized on or near the plasma membrane, which was visualized as a cell contour, were counted.

### Reagents

TG and 2-APB were obtained from Sigma. ML-9 was purchased from Tocris Biosciences. All compounds were dissolved in DMSO. Fura-2 was obtained from Molecular Probes.

### Statistical analysis

Statistical analysis was performed with Prism version 5.02 software (GraphPad, San Diego, CA, USA). All pooled data are expressed as mean ± SD. One-way analysis of variance (ANOVA) was performed to analyze sets of data. Tukey's *post hoc* test was used to determine statistically significant differences among groups. The degree of significance *vs*. control is indicated by asterisks: *p<0.05, **p<0.01, ***p<0.001 (ns, not significant, *p*>0.05).

## Supporting Information

Figure S1 **Confocal analysis of neurons co-transfected with YFP-STIM1 or YFP-STIM2 and ORAI1**. Representative overlay images from three independent experiments of neurons co-expressing ORAI1 and YFP-STIM or YFP-STIM2 in the presence of 2 mM extracellular Ca^2+^ before store depletion (top panels) 10 min following treatment with 2 µM TG in 0.5 mM EGTA (center panels) or 2 mM EGTA alone (bottom panels). The cells expressing YFP-STIM (green) and ORAI1 were then stained with ORAI1 antibody (red). Neurons were analyzed using a Leica confocal microscope, and images represent 0.25 µM thick confocal scans.
(TIF)

Figure S2 **Analysis of constitutive calcium entry in transfected cortical neurons.** Cytosolic Ca^2+^ measurements were performed in cells overexpressing YFP-STIM1 ± ORAI1 or YFP-STIM2 ± ORAI1 or in nontransfected (NT) cells. The experiments started in the presence of 2 mM CaCl_2_, followed by transfer to Ca^2+^-free medium. A buffer was then supplemented with 2 mM CaCl_2_ for 5 min to monitor intracellular Ca^2+^ restoration (**A**). Finally, 50 µM 2-APB was added (**B**). The measurements were performed relative to the average [Ca^2+^]_i_ recorded in Ca^2+^-free medium (200–400 s of experiment) (**A**) or relative to the average [Ca^2+^]_i_ recorded in Ca^2+^-rich medium (**B**) normalized to 1. Raw traces are shown in Figure 6A.
(TIF)
