# Supplementary figures and images for: Differential Roles for STIM1 and STIM2 in Store-Operated Calcium Entry in Rat Neurons
Source: PLoS One. 2011 Apr 26;6(4):e19285. doi: 10.1371/journal.pone.0019285 (PMC3082561; doi:10.1371/journal.pone.0019285)

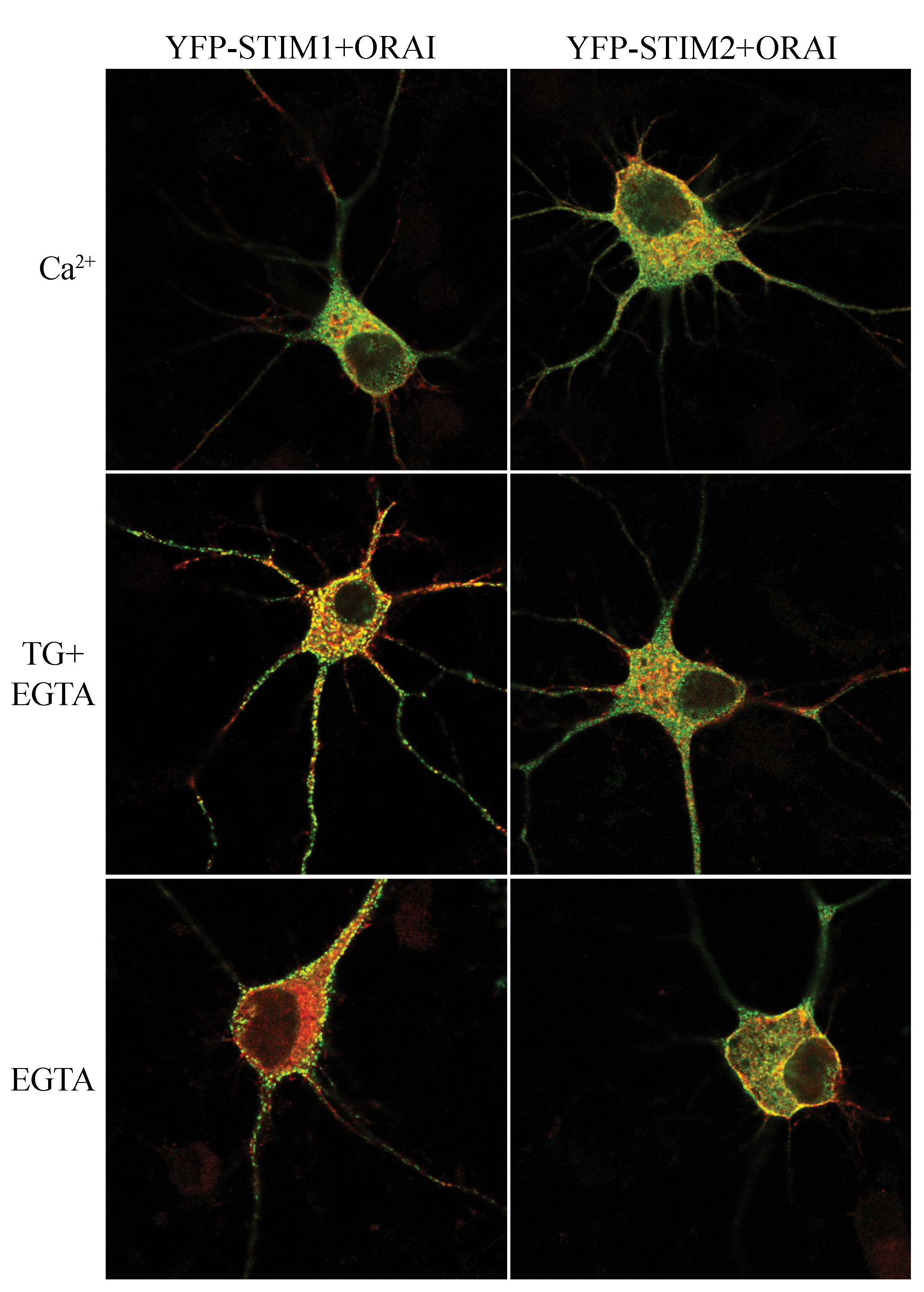

Supplement: Figure S1 — Confocal analysis of neurons co-transfected with YFP-STIM1 or YFP-STIM2 and ORAI1. Representative overlay images from three independent experiments of neurons co-expressing ORAI1 and YFP-STIM or YFP-STIM2 in the presence of 2 mM extracellular Ca2+ before store depletion (top panels) 10 min following treatment with 2 µM TG in 0.5 mM EGTA (center panels) or 2 mM EGTA alone (bottom panels). The cells expressing YFP-STIM (green) and ORAI1 were then stained with ORAI1 antibody (red). Neurons were analyzed using a Leica confocal microscope, and images represent 0.25 µM thick confocal scans. (TIF) [file pone.0019285.s001.tif]

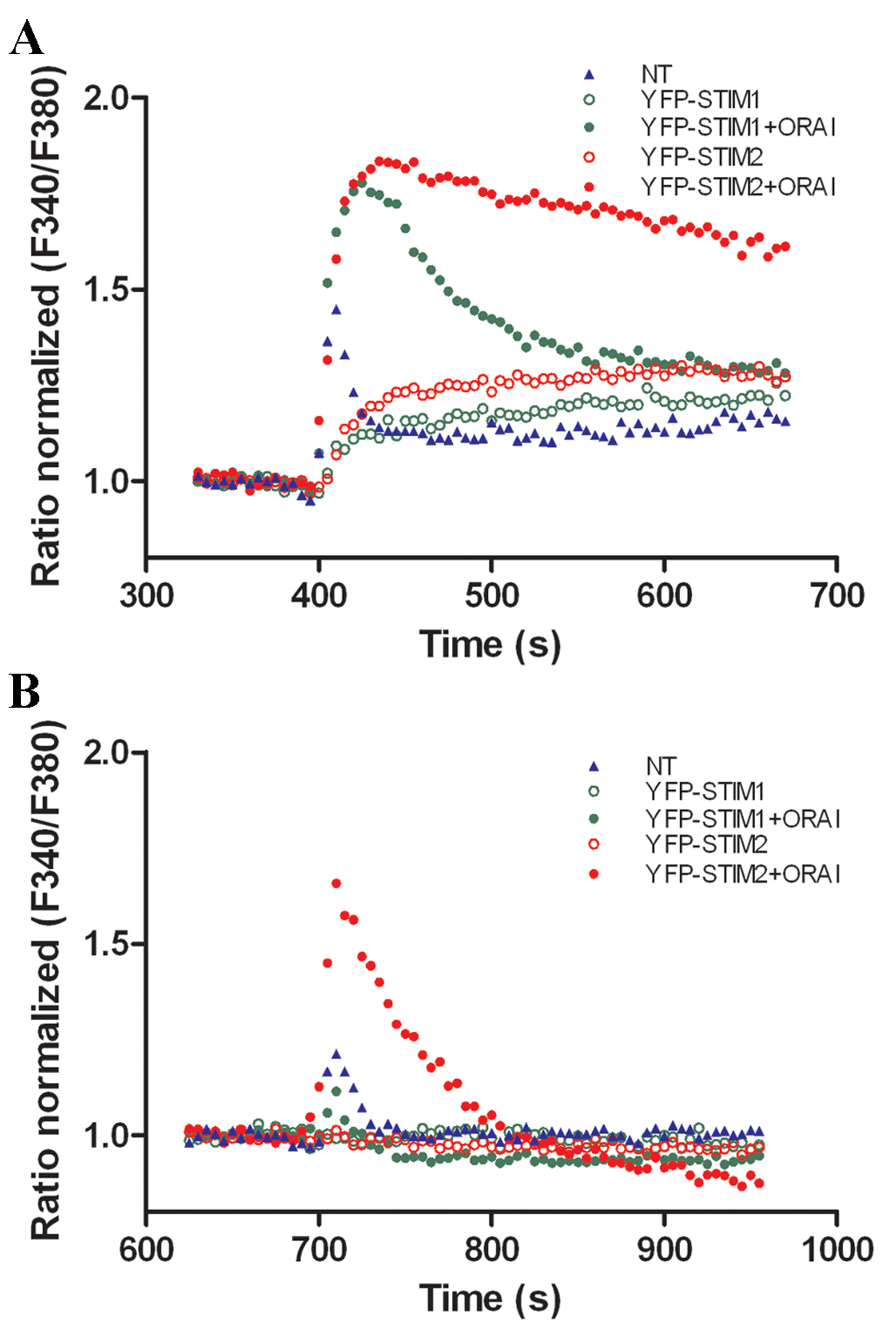

Supplement: Figure S2 — Analysis of constitutive calcium entry in transfected cortical neurons. Cytosolic Ca2+ measurements were performed in cells overexpressing YFP-STIM1 ± ORAI1 or YFP-STIM2 ± ORAI1 or in nontransfected (NT) cells. The experiments started in the presence of 2 mM CaCl2, followed by transfer to Ca2+-free medium. A buffer was then supplemented with 2 mM CaCl2 for 5 min to monitor intracellular Ca2+ restoration (A). Finally, 50 µM 2-APB was added (B). The measurements were performed relative to the average [Ca2+]i recorded in Ca2+-free medium (200–400 s of experiment) (A) or relative to the average [Ca2+]i recorded in Ca2+-rich medium (B) normalized to 1. Raw traces are shown in Figure 6A. (TIF) [file pone.0019285.s002.tif]
